# Clinical Value of Three Combined Ultrasonography Modalities in Predicting the Risk of Metastasis to Axillary Lymph Nodes in Breast Invasive Ductal Carcinoma

**DOI:** 10.3389/fonc.2021.715097

**Published:** 2021-09-22

**Authors:** Qing Zhang, Enock Adjei Agyekum, Linna Zhu, Lingling Yan, Lei Zhang, Xian Wang, Liang Yin, Xiaoqin Qian

**Affiliations:** ^1^Department of Ultrasound, Jiangsu University Affiliated People’s Hospital, Zhenjiang, China; ^2^Department of Breast Surgery, Jiangsu University Affiliated People’s Hospital, Zhenjiang, China; ^3^School of Medicine, Jiangsu University, Zhenjiang, China

**Keywords:** breast cancer, axillary lymph node, conventional ultrasound, ultrasound elastography, percutaneous contrast-enhanced ultrasound, breast-invasive ductal carcinoma, sentinel lymph node

## Abstract

**Objective:**

The present study aimed to assess the clinical value of conventional ultrasound (C-US), ultrasound elastography (UE), percutaneous contrast-enhanced ultrasound (P-CUES), and the combination of these three ultrasonography modalities for evaluating the risk of axillary lymph node (ALN) metastasis in breast invasive ductal carcinoma (IDC).

**Methods:**

This retrospective analysis included 120 patients with pathologically confirmed IDC who underwent sentinel lymph node biopsy (SLNB) or axillary lymph node dissection (ALND). Based on the gold standard of postoperative pathology, ALN pathology results were evaluated and compared with findings obtained using C-US, UE, P-CUES, and the three modalities combined.

**Results:**

(1) There was a statistically significant difference between the histological grade of the tumor and the pathological condition of ALNs. (2) The difference between C-US parameters and UE score were statistically significant. The accuracy of P-CEUS localization of SLNs was 100% (96/96) when compared with localization guided by methylene blue. The difference in the distribution of the four SLN enhancement patterns was statistically significant. (3) The sensitivity, specificity, positive predictive value, and negative predictive value of C-US and UE were 75%, 71%, 58%, and 89%, and 71%, 72%, 50%, and 86%, respectively. The sensitivity, specificity, positive predictive value, and negative predictive value of P-CUES were 91%, 82%, 78%, 92%, respectively. When all three modalities were combined, the sensitivity, specificity, positive predictive value, and negative predictive value were 94%, 89%, 86%, and 95%, respectively. In the detection of ALN metastasis, there was a good correlation between histopathological results and evaluations based on the three combined ultrasonography modalities (kappa: 0.82, p<0.001).

**Conclusions:**

When compared to C-US, UE, or P-CEUS alone, the combination of the three ultrasonography modalities was found to be superior in distinguishing metastatic and non-metastatic ALNs. This combined strategy may aid physicians in determining the most appropriate approach to ALN surgery as well as the prognosis of breast IDC.

## 1 Introduction

Breast cancer is among the tumors with the highest incidence in women and usually causes the most serious harm among female patients. The most important factors determining prognosis include tumor size, histological grade, hormonal receptor (HR) status, human epidermal growth factor receptor 2 (HER-2) status, axillary lymph node (ALN) involvement, and metastasis (1, 2). ALNs are the most common metastatic site of breast cancer and an important factor in guiding the treatment and management of breast cancer (3, 4), which also plays an important role in patient prognosis. Sentinel lymph node biopsy (SLNB) has gradually replaced traditional axillary lymph node dissection (ALND) and has become the standard technique for diagnosing the pathological status of ALNs in breast cancer. SLNB can significantly reduce complications such as pain, swelling of the affected limb, and lymphatic reflow disorder when compared with ALND.

The SLNs are the hypothetical first LN or group of nodes draining a local tissue or primary cancer. Given that LN metastasis is one of the most critical prognostic factors, the status of SLNs in patients with breast cancer is critical. Currently, magnetic resonance imaging (MRI), ultrasonography, and other imaging methods are mostly used for preoperative ALN diagnosis. However, MRI is expensive and requires long imaging times. Ultrasound examination is non-invasive, non-ionizing, and inexpensive, it can provide real-time information, and is widely available and flexible for use in clinical practice when compared to other imaging modalities.

To aid clinicians in making the most appropriate preoperative and surgical decisions, the present study aimed to assess the diagnostic value of the individual application of conventional ultrasound (C-US), ultrasound elastography (UE), and percutaneous contrast-enhanced ultrasound (P-CEUS), as well as the combination of these three US modalities (3C-USMs), for investigating ALNs in breast IDC. Few studies have investigated the ability of the combination of the three ultrasonography modalities to predict the pathological condition of ALNs prior to surgery.

## 2 Patients and Methods

### 2.1 Patients

The study included 120 women with breast IDC who underwent surgical treatment at Jiangsu University Affiliated People’s Hospital between May 2019 and April 2021 of which 35 of them having axillary lymph node metastasis and receiving neo-adjuvant chemotherapy after surgery. The inclusion criteria were as follows: (a) breast IDC confirmed *via* postoperative pathology; (b) age >18 years; (c) lack of palpable axillary lymph nodes;(d) performance of preoperative C-US, UE and a signed consent form for conducting a P-CEUS examination to evaluate the status of ALNs; (e) lack of palpable axillary lymph nodes; (f) qualified for SLNB. Exclusion criteria were as follows: (a) pathological types other than IDC, due to the low incidence rate and the small number of cases; (b) presence of inflammatory breast cancer; (c) history of previous axillary surgery or chemotherapy; (d) pregnancy or allergy to ultrasound contrast agents. This study was approved by the Ethics Committee of Jiangsu University Affiliated People’s Hospital, and all patients provided written informed consent.

### 2.2 Ultrasound Examination

#### 2.1.1 Ultrasound Imaging Instruments

A Philips EPI Q5 color Doppler ultrasound imaging instrument was used to perform C-US, UE, and P-CUES (using a 12-5 MHZ and 9-3MHZ linear-array transducer). Low mechanical index values (MI=0.08) were applied to reduce the destruction of the contrast agent.

#### 2.2.2 C-US Examination

The patient’s posture was adjusted to the supine position with arms raised so that the bilateral mammary glands and axilla were fully exposed, and multi-section scanning was performed horizontally and vertically along the direction of the axillary vein, including the ALN at the I–III level of the ipsilateral axillary vein. C-US was used to visualize the lymphatic hilum, short/long axis of LNs (L/T), cortical thickness, and blood flow signal of the internal and peripheral lymph. When there were multiple abnormal LNs, the number of abnormal LNs and all abnormal LN images were recorded and saved.

#### 2.2.3 UE Examination

The ALNs were selected to display the best section, and after further adjusting the depth, focus, and gain of the image to obtain a satisfactory two-dimensional image, the probe was fixed, and the UE was switched to side-by-side mode. The sampling frame was larger than the lesion range (generally 2-3 times the lesion size), and the UE was observed continuously. During the examination, the patients were instructed to hold their breath intermittently in order to obtain the appropriate images. Slight pressure was applied to maintain contact of the probe with the skin. The hardness of the lesions was closely observed using the real-time dual mode of the elastic and greyscale maps, and the relative hardness was analyzed with blue, green, and red colors.

#### 2.2.4 P-CEUS Examination

The second-generation contrast agent SonoVue (Bracco SpA, Milan, Italy) was used in this study. The SonoVue freeze-dried powder, which contained sulfur hexafluoride-filled microbubbles, was dissolved in 5 ml of saline (NaCl 0.9%) before injection. P-CEUS was performed after subdermal injection of the ultrasound contrast agent (SonoVue, total dose of 2.0 ml) around the areola at 12, 3, 6, and 9 o’clock of the breast and gently massaged for 5–10 s to promote the flow of contrast agent to the lymphatic vessels. Enhanced LNs were detected by moving the probe along the channels. The first or first group of enhanced LNs was considered to represent the SLN along the enhanced lymphatic vessels. Live dual images were used to confirm the presence of an architecturally defined SLN. If the lymphatic channel or lymph node was not detected successfully, one or two additional injections were performed. Once identified, the lymphatic vessels and associated SLNs were labeled on the skin surface. The number, location, distance to the body surface, and enhancement of the SLNs were documented.

### 2.3 Criteria for Diagnosis and Image Analysis

#### 2.3.1 C-US Criteria for Evaluating ALN Status

The criteria for evaluating ALN status on C-US were as follows: L/T <2, disappearance of lymphatic hilum structure, uneven thickening of the cortex (>3 mm), heterogeneous internal echo, and mixed peripheral blood flow signal. When at least two of these abnormal indicators were observed, C-US was considered to suggest LN metastasis (5, 6).

#### 2.3.2 UE Criteria for Evaluating ALN Status

The elastography data sets were analyzed qualitatively using the elasticity score, which is a color-coded 5-stage score (7). A score of 1 point indicates that the diseased tissue and surrounding tissue are completely covered in green. A score of 2 points indicates that the lesion area contains mixed blue and green areas, with green as the dominant color. A score of 3 points indicate that the lesion area is mainly blue with a surrounding green area. A score of 4 points indicates that the lesion area is completely covered by blue. A score of 5 points indicates that the lesion area is completely covered by blue, and that a small part of the surrounding tissue is also blue. Green indicates the texture of the lesion is soft, blue means the texture of the lesion is hard. Lesions with scores of 1–3 are assumed to be non-metastatic ALNs, while those with scores of 4 or 5 are suspected to be metastatic ALNs (8, 9).

#### 2.3.3 P-CUES Criteria for Evaluating SLN status

**Figure 1:**
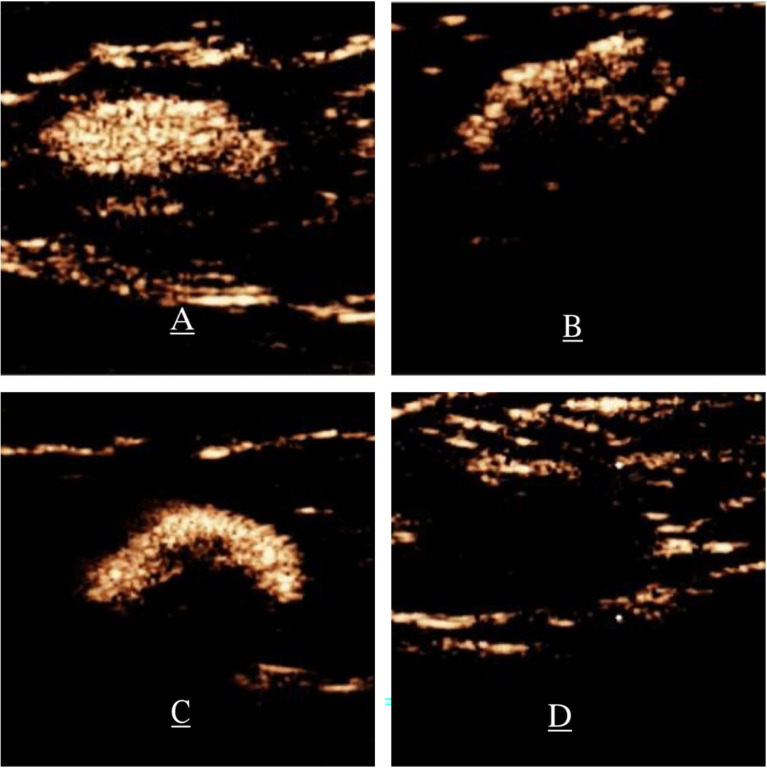
Patterns of enhancement on P-CEUS. **(A)** Type I, homogenous enhancement. **(B)** Type II, heterogeneous enhancement, significantly enhanced parenchymal areas of focal hypoperfusion or non-perfusion. **(C)** Type III, complete or incomplete circumferential ring enhancement and low or no central enhancement. **(D)** Type IV, slight enhancement or no enhancement. P-CEUS: percutaneous contrast-enhanced ultrasound.

The pattern of SLN enhancement was divided into the four types: homogeneous enhancement (Type I, **Figure 1A**), heterogeneous enhancement with a mixture of high and low enhancement (Type II, **Figure 1B**), complete or incomplete peripheral enhancement with low or no central enhancement (Type III, **Figure 1C**), and slight enhancement or no enhancement (Type IV, **Figure 1D**) (10, 11). Type I predicts that the SLN is a non-metastatic ALN, while type II, III, and IV SLNs are predicted to be metastatic ALNs (12).

#### 2.3.4 Operation and Criteria for Pathological Diagnosis of ALNs

After tracheal intubation anesthesia and routine disinfection, sterile drapes were spread, and 2 ml of methylene blue was injected around the areola and massaged for 5–10 min for intradermal mapping. A curved surgical incision was made in the axillary area near the lateral border of the pectoralis major muscle. The skin and subcutaneous tissues were cut to identify blue-stained lymph vessels and LNs, and the location, number, and morphology were observed. For example, the blue-stained LN body surface projection was consistent with the location of the LN marked on the P-CEUS body surface, and it was confirmed to represent the same LN. After removal of the LN, rapid pathology was sent. Some studies (13) have suggested that, if an appropriate technique is used to determine micro-metastasis or isolated cell metastasis, there is a decreased need for SLN sampling and ALND, thereby reducing the risk of complications and morbidity. ALNs were considered negative if the pathological results indicated no metastasis (including isolated cell metastasis, micro-metastasis). If the pathological results indicated macro-metastasis, the LNs were considered positive, ALND was performed, and the number of ALNs with macro-metastasis was counted after the operation.

The above ultrasonographic techniques and the pathologic analyses were performed by two experienced sonographers using the double-blind method. If there were some disagreements, consensus was reached through mutual consultation. The pathological results were used as the gold standard. When even one of the three combined ultrasonography modalities diagnosed an abnormal LN, LN metastasis was suspected.

### 2.4 Statistical Analysis

The IBM SPSS statistical software (version 21.0, IBM Corp., Armonk: NY) was used for data analysis. T-tests were used to evaluate continuous variables such as average age and the maximum diameter of the mass. The chi-square test or Fisher’s exact probability method was used for measurable variables, including histological grade, status of ER and HER2, L/T, lymphatic hilum structure, cortex, internal echo, blood flow signal, UE Score, and enhancement mode. The kappa value was used to evaluate consistency between the different ultrasound methods and pathological diagnosis. The rapid intraoperative pathological results were used as the gold standard to assess ALNs status, and the sensitivity, specificity, accuracy, false negative rate, and false positive rate for C-US, UE, P-CEUS, and the combined method for the diagnosis of ALN metastasis. *P* <0.05 was considered statistically significant.

## 3 Results

### 3.1 Comparison of ALN Metastasis Risk Based on Clinical Characteristics

A total of 120 female patients were pathologically diagnosed with breast IDC before treatment. Their average age was 50.5 ± 9.6 years, and the age range was 32–77 years. There was a statistically significant difference between the histological grade of the tumor and the pathological condition of ALNs. However, there were no significant differences in the average age, maximum diameter of the tumor, or the status of ER or HER2 between (**Table 1**).

**Table 1:** Comparison of clinicopathologic factors and ALN metastasis in patients with breast invasive ductal carcinoma.

Variable	Number	Pathological condition of ALNs		P value	x^2^ value
		Metastatic	Non-metastatic		
Cases	120	35	85		
Average age (y)	50.5 ± 9.6	51.2 ± 9.3	50.0 ± 9.8	0.81	
Maximum diameter (cm)		3.1 ± 2.0	2.2 ± 0.8	0.07	
Histological grade				6.45	0.04
Grade I	20	12	8		
Grade II	70	40	30		
Grade III	30	25	5		
ER				0.44	0.59
(+)	85	75	10		
(-)	35	29	6		
HER2				0.57	0.31
(+)	22	18	3		
(-)	98	79	19		

ALN, axillary lymph node; ER, Estrogen receptor; HER2: Human epidermal growth factor receptor 2.

### 3.2 Comparison of ALN Metastasis Risk Predicted Based on C-US, UE Score, and P-CEUS Parameters

#### 3.2.1 Comparison Between C-US and Postoperative LN Pathological Results

Among metastatic LNs, 86% exhibited L/T <2, 86% exhibited an absence of the lymphatic hilum, 71% exhibited thickening of the cortex, 77% exhibited a heterogeneous internal echo, and 83% exhibited peripheral and mixed blood flow signals. C-US revealed the following for metastatic ALNs: disappearance of lymphatic hilum structure, thickening of the cortex (>3 mm), change in the proportion of uniform echo, L/T <2, and peripheral or mixed blood flow signals. These differences were statistically significant (P <0.05). Twenty abnormal LNs were detected by C-US. (The values of C-US parameters for predicting the risk of ALN metastasis are shown in **Table 2**).

**Table 2:** Comparison of LN metastasis risk predicted by C-US, UE score, and P-CEUS parameters.

Variables	Pathological type		P value	x^2^
	Positive	Negative		
L/T			0.00	76.48
L/T <2	30	5		
L/T >2	5	80		
Lymphatic hilum structure			0.00	61.01
Presence	5	75		
Loss	30	10		
Cortex			0.00	32.27
Normal	10	70		
Thick	25	15		
Internal echo			0.00	44.89
Homogeneous	8	73		
Heterogeneous	27	12		
Blood flow signal			0.00	85.22
Normal	2	73		
None	2	1		
Peripheral	12	2		
Central	2	6		
Mixed	17	3		
UE Score			0.00	58.71
1	2	12		
2	2	56		
3	8	10		
4	12	4		
5	11	3		
Enhancement pattern			0.00	90.82
Type I	2	78		
Type II	5	2		
Type III	23	3		
Type IV	5	2		

LN, lymph node; L/T, short/long axis of lymph nodes; C-US, conventional ultrasound; ALN, axillary lymph node; UE, ultrasound elastography; P-CEUS, percutaneous contrast-enhanced ultrasound.

#### 3.2.2 Comparison Between UE and Postoperative LN Pathological Results

In 66% of all metastatic LNs, the UE score was 4–5 points. UE scores for 92% of non-metastatic ALNs ranged from 1–3 points. Fifteen abnormal LNs were detected by UE. The difference was statistically significant (**Table 2**).

#### 3.2.3 Comparison Between P-CEUS and Postoperative LN Pathological Results

The contrast agent was injected subcutaneously around the areola, reaching the SLN within a time frame of 15–35 s, and enhancement of the ALN edge began to appear after 35 s. Full LN enhancement was observed within 2 min, and the contrast agent could be cleared after 3–6 min, which allowed the sonographers to locate the SLN. It is worth noting that P-CEUS shows SLNs rather than all ALNs. In P-CEUS body surface localization, all SLNs were stained with methylene blue, and the accuracy of P-CEUS localization of SLNs was 100% (96/96) when compared with localization guided by methylene blue. P-CEUS detected 170 SLNs in 120 patients. Among these SLNs, 30 were metastatic, and 140 were non-metastatic. In this study, the difference in the distribution of the four SLN enhancement patterns was statistically significant. Type I predicts that the SLN is non- metastatic, while type II, III, and IV SNLs were predicted to be metastatic (**Table 2**).

### 3.3 Comparison of ALN Metastasis Risk Predicted by the Combination of the Three Ultrasound Modalities

The combined ultrasound procedure took an average time of 15.50±3.61 minutes. The pathological results (the gold standard) for ALN metastasis in breast IDC were consistent with those obtained using C-US (kappa =0.51, P=0.000), UE (Kappa=0.37, P=0.000), and P-CEUS (Kappa=0.72, P =0.000). The pathological results for ALN metastasis were also consistent with those obtained using the combination of the three ultrasonography modalities (Kappa=0.82, P =0.000), which had a higher kappa value than that of each single modality. Pathological analysis identified 38 metastatic SLNs, while the combined modalities identified 33 such LNs. This number was greater than that for each individual ultrasound modality. The sensitivity, specificity, false negative rate, false positive rate, and diagnostic accuracy of the combined ultrasonography modalities for predicting ALN metastasis of breast cancer were also higher than those for the individual modalities (**Table 3**).

**Table 3:** Diagnostic ability of C-US, UE, P-CEUS, and the three modalities combined.

Parameters	C-US	UE	P-CEUS	Combination of the three ultrasonography modalities
Sensitivity	82%	72%	82%	89%
Specificity	75%	71%	91%	94%
Positive predictive value	58%	50%	78%	86%
Negative predictive value	89%	86%	92%	95%
False positive rate	25%	29%	9%	6%
False negative rate	17%	29%	17%	11%
Accuracy	77%	71%	88%	92%

C-US, conventional ultrasound; ALN, axillary lymph node; UE, ultrasound elastography; P-CEUS, percutaneous contrast-enhanced ultrasound.

## 4 Discussion

Among all malignant tumors in women, breast cancer has the highest incidence (14). Early breast cancer has no obvious symptoms, causes serious physical and mental harm to women, and poses a serious threat to their lives. Axillary lymph node status is the most important prognostic factor in breast cancer survival. As a result, knowing the status of axillary lymph nodes is critical for determining prognosis, staging, and treatment planning. With the help of ultrasound imaging, a clear determination of whether ALNs are malignant or benign ensures proper staging (15). Successful staging, in turn, affects prognosis and survival by ensuring the appropriate treatment plan is chosen. Therefore, we studied the value of C-US, UE, P-CEUS, and the combination of the three modalities in preoperative assessment of the status of axillary lymph nodes with breast IDC.

In this study, conventional pathological analysis identified 35 cases with metastasis and 85 cases without metastasis. In patients with clinical pathologic factors (including age, tumor size, tumor grade, and ER and HER2 status), higher degrees of malignancy were associated with an increased risk of ALN metastasis. Some previous studies (16) and clinical practitioners have also noted a positive correlation between lymph node metastasis and pathological grade.

Imaging features on C-US indicated that metastatic ALNs exhibited L/T <2, disappearance of the lymphatic hilum structure, cortical thickening, heterogeneous echogenicity, and peripheral or mixed blood flow signals. The sensitivity of C-US was 82%, while its specificity was 75%. Previous studies have similarly reported C-US sensitivity and specificity values of 80% and 71%, respectively (17). This may be closely related to the pathological basis of LNs: (a) Normal LNs are often characterized by lymphatic hilum structures (e.g., hyperechoic medullary tissue), homogeneous echogenicity, and portal blood flow signal; however, when LN metastasis first begins, invasion first occurs in the marginal lymph sinus in the subscapular area through the input lymphatic vessels, resulting in abnormal proliferation of the marginal sinus, which gradually widens and thickens the cortex. After invasion of the medulla, the medulla begins to narrow or even disappear, resulting in disappearance of the lymphatic hilum structure, and the internal echo becomes uneven (5, 18). (b) The normal LN tissue is replaced by the tumor nest tissue, and the original LN blood vessels are distorted or proliferate abnormally, transforming into abnormal tumor blood vessels. Although C-US is rather successful in predicting ALN status, some studies have shown that C-US cannot distinguish which of the ALND are SLNB (19)

Guo et al. (20) reported that the sensitivity and specificity of UE for the diagnosis of ALN metastasis were 74.0% and 72.1% when using a UE cutoff score of 3, respectively. However, in our study, most UE scores for metastatic LNs were 4–5 points, and the sensitivity and specificity of UE were 72% and 71%, respectively. This may be due to the process involved in the transition from normal ALNs to metastatic LNs, which involves the proliferation of connective tissue, an increase in collagen fibers, and abnormalities in tissue composition and structure inside the tumor. The hardness of the tumor increases, causing the elasticity of the tumor to increase (21).

However, UE examination of ALNs cannot always be used to obtain valid information due to factors such as uneven skin, deep position, and adjacency to large blood vessels, overlapping elasticity coefficient, or operator’s technique (22, 23). In some cases, ALNs become soft when they undergo liquefaction and necrosis, which leads to false negatives.

It’s worth noting that SLNB has gradually replaced the traditional ALND as the gold standard for determining the pathological status of ALNs in breast cancer. As a result, it is critical to accurately locate and characterize the SLNs prior to surgery. P-CEUS was used in this study to mark the lymphatic vessels and associated SLNs on the skin surface, to determine which ALND are SLNB, and to document the number, location, distance to the body surface, and enhancement of the SLNs. This study discovered that all SLNs marked by P-CEUS preoperatively were stained with methylene blue, and that the accuracy of P-CEUS localization of SLNs was 100% (96/96). The difference in enhancement patterns between metastatic and non-metastatic SLN was statistically significant, implying that P-CEUS can be used to assess the status of SLNs prior to surgery. C-US, UE, P-CEUS, and the combination of the three modalities can effectively evaluate the status of ALNs and guide ALNs biopsy improving sensitivity, specificity, and accuracy of location and characterization of ALNs.

In this study, 140 of all 142 LNs exhibiting homogeneous hyperenhancement were benign, suggesting that homogeneous hyperenhancement is the typical enhancement pattern for non-metastatic LNs. Metastatic LNs often exhibit heterogeneous enhancement, slight enhancement, or no enhancement. The sensitivity, specificity, positive predictive value, and negative predictive value were 82%, 91%, 78%, and 92%, which was consistent with the findings of relevant studies (24–26).

When a SLN metastasizes, the microbubbles first enter the marginal lymph sinus through the input lymphatic vessels and then infiltrate into the parenchyma with the tumor cells. After the contrast agent enters the SLNs through the lymphatic vessels, lymphatic drainage is blocked due to the infiltration of tumor cells, which may lead to marginal defects and heterogeneous enhancement. In this study, type II or III enhancement was observed in eight cases; however, pathological analysis in these cases indicated that the SLNs were non-metastatic. The reasons for false-positive results may be as follows: (a) SLNs in breast IDC may include lymphatic follicular hyperplasia, lymphatic sinus dilatation, and reactive hyperplasia. Although previous studies have shown that P-CEUS can effectively delineate lymphatic vessels and SLNs, tumor metastasis should be considered in cases with no enhancement or heterogeneous enhancement of the LNs (27, 28).

However, some benign lesions such as lymphatic follicular hyperplasia, lymphatic sinus dilatation, and chronic inflammation may also block lymphatic drainage, resulting in retention or slow enhancement of contrast agent in lymphatic parenchyma tissue and heterogeneous enhancement, which can easily lead to a misdiagnosis of metastatic LNs. (b) In this study, six SLNs exhibited heterogeneous enhancement; however, pathological results indicated the presence of adipose tissue in the LNs. This may be due to excessive fat or wrapping around the SLN. (c) There was another SLN with granulomatous tissue that exhibited heterogeneous enhancement. Postoperative pathology confirmed that the tumor thrombus partially obstructed lymphatic vessels. In this case, the contrast agent could not enter the LNs, and no SLNs were found. (d) Lymphatic flow is positively correlated with regional tissue pressure and negatively correlated with lymphatic reflux pressure. The lymphatic wall is composed of smooth muscle and is characterized by spontaneous rhythmic contractions. If rhythmic massage is greater than the spontaneous contraction rhythm of the initial lymphatic vessels, lymphatic flow will increase. If the operator injection site and direction of contrast agent, massage time, and massage intensity are not sufficient, this will prevent the contrast agent from completely reaching the LN or cause microbubble destruction to different degrees, which will also affect the enhancement of LNs. P-CEUS examination has an important diagnostic value for SLN properties in patients with breast IDC. P-CEUS is also advantageous in that it is non-invasive, utilizes non-ionizing radiation, and can usually be performed quickly. The shape of the sentinel lymphatic vessels and the position, size, and enhancement pattern of LNs can be observed in real time using P-CEUS; however, the positive predictive value is low, which affects the identification of metastatic ALNs.

This study had some limitations. Firstly, only one pathological type of breast cancer was included in this study, leading to selection bias. Secondly, this study was a single-center and retrospective analysis, which makes it impossible to comprehensively evaluate the diagnostic efficacy of three combined ultrasound modalities. Also in this study, the rapid intraoperative pathological results were used as the gold standard to assess ALNs status, so, the suspicious LNs before surgery were not biopsied. Also, in suspicious LNs, no markers were placed. However, based on the findings of this study, our team has begun to perform preoperative puncture biopsy on suspicious LNs, and if the pathological findings indicate metastatic LNs, we will recommends that the patient receives neo-adjuvant chemotherapy first. We also intend to place markers in suspicious LNs in future studies.

In conclusion, in the analysis of ALNs in patients with breast IDC, the combination of the three ultrasonography modalities yielded higher sensitivity, specificity, positive predictive value, negative predictive value, and accuracy than each individual modality. Compared with ALND and SLNB, these imaging methods are non-invasive and exhibit good diagnostic performance, indicating that they can be useful for selecting appropriate treatment strategies and evaluating prognosis in patients with breast IDC. Advancements in imaging methods and verification of the present findings will lead to decreased rates of ALND. Future studies should aim to clarify the contribution of the axilla to surgical management based on this combination of images.

## Data Availability Statement

The raw data supporting the conclusions of this article will be made available by the authors, without undue reservation.

## Ethics Statement

The studies involving human participants were reviewed and approved by Ethics Committee of Jiangsu University Affiliated People’s Hospital. Written informed consent for participation was not required for this study in accordance with the national legislation and the institutional requirements.

## Author Contributions

QZ, EA, and LLY contributed to the conception and design of the study. XW organized the database. QZ, LiZ, and EA performed the statistical analysis. QZ and EA wrote the first draft of the manuscript. LY, XQ and LeZ wrote sections of the manuscript. All authors contributed to the article and approved the submitted version.

## Funding

This study was financially supported by National Natural Science Foundation of China (Project No.:81971629) and Research Project of Jiangsu Maternal and Child Health Association (Grant No. FYX202004).

## Conflict of Interest

The authors declare that the research was conducted in the absence of any commercial or financial relationships that could be construed as a potential conflict of interest.

## Publisher’s Note

All claims expressed in this article are solely those of the authors and do not necessarily represent those of their affiliated organizations, or those of the publisher, the editors and the reviewers. Any product that may be evaluated in this article, or claim that may be made by its manufacturer, is not guaranteed or endorsed by the publisher.
